# Levels of (1→3)-β-D-glucan, *Candida *mannan and *Candida *DNA in serum samples of pediatric cancer patients colonized with *Candida *species

**DOI:** 10.1186/1471-2334-10-292

**Published:** 2010-10-06

**Authors:** Eiman Mokaddas, Mona HA Burhamah, Zia U Khan, Suhail Ahmad

**Affiliations:** 1 Department of Microbiology, Faculty of Medicine, Kuwait University, Safat, 13110. P. O. Box 24923, Kuwait; 2 NBK Cancer Ward, Al-Sabah Hospital, Kuwait

## Abstract

**Background:**

Surveillance cultures may be helpful in identifying patients at increased risk of developing invasive candidiasis. However, only scant information exists on the effect of *Candida *colonization on serum levels of diagnostic biomarkers. This prospective surveillance study determined the extent of *Candida *colonization among pediatric cancer patients and its possible impact on serum levels of (1-3)-β-D-glucan (BDG), *Candida *mannan and *Candida *DNA.

**Methods:**

A total of 1075 swabs originating from oropharynx (n = 294), nostrils (n = 600), rectum (n = 28), groin (n = 50), ear (n = 54), and axilla (n = 49) of 63 pediatric cancer patients were cultured for the isolation of *Candida *spp. Patients yielding *Candida *spp. from any sites were considered as colonized. Serum samples were collected from patients at the time of first surveillance culture for detection of BDG by Fungitell kit and *Candida *mannan by Platelia *Candida *Ag. *Candida *DNA was detected by using panfungal primers and identification was carried out by using species-specific primers and DNA sequencing.

**Results:**

Seventy-five (7.6%) swab cultures from 35 (55.5%) patients yielded *Candida *spp. These isolates included *C. albicans *(n = 62), *C. dubliniensis *(n = 8), *C. glabrata *and *C. tropicalis *(n = 2 each) and *C. krusei *(n = 1). Eleven patients were colonized at three or more sites. Eight of 36 serum samples from 6 colonized patients yielded BDG values higher than the currently recommended cut-off value of ≥80 pg/ml. However, none of the serum samples yielded *Candida *mannan levels ≥0.5 ng/ml and PCR test for *Candida *DNA was also negative in all the serum samples of colonized patients. During the study period, only two colonized patients subsequently developed candidemia due to *C. tropicalis*. Besides positive blood cultures, *C. tropicalis *DNA, BDG and *Candida *mannan were also detected in serum samples of both the patients.

**Conclusions:**

The present study demonstrates that while mucosal colonization with *Candida *species in pediatric cancer patients is common, it does not give rise to diagnostically significant levels of *Candida *mannan or *Candida *DNA in serum specimens. However, BDG values may be higher than the cut-off value in some pediatric patients without clinical evidence of invasive *Candida *infection. The study suggests the utility of *Candida *mannan or *Candida *DNA in the diagnosis of invasive candidiasis, however, the BDG levels in pediatric cancer subjects should be interpreted with caution.

## Background

The incidence of fungal infections among cancer patients has shown a steady increase in recent years [1-3]. This may partly be attributed to the use of more aggressive chemotherapeutic regimens, resulting in more prolonged survival of these immunosuppressed patients while they continue to remain vulnerable to invasive fungal infections [4,5]. Although establishing an early diagnosis for invasive mycoses is ideal for timely administration of specific antifungal therapy, it invariably gets delayed due to want of culture or histopathologic evidence [5]. Strategies are now being evolved to identify a subgroup of high-risk patients where prophylactic or empirical therapeutic approach could be used for preventing development of invasive fungal infections [6]. Recently, Maertens *et al. *[7] proposed a preemptive approach based on radiologic and other surrogate markers for the early diagnosis of invasive mycoses in high-risk patients. Surveillance cultures for determining the *Candida *colonization index in high-risk patients may be helpful in identifying patients at increased risk of invasion and hematogenous dissemination [8-11]. However, only scant information is available on the effect of *Candida *colonization on the serum levels of BDG, *Candida *mannan or *Candida *DNA [12-14]. In the present communication, we report results of *Candida *colonization among hospitalized pediatric cancer patients and its possible impact on serum levels of BDG, *Candida *mannan, and *Candida *DNA.

## Methods

### Study population

The study was carried out in a tertiary care Pediatric Cancer Ward, Al-Sabah Hospital, Kuwait between July 1, 2007 to December 31, 2008. Sixty-three cancer patients, 57 (90%) with acute lymphoblastic leukemia (ALL) and 6 (10%) with acute myeloid leukemia (AML) were followed-up by weekly surveillance cultures for varying periods for assessing the extent of *Candida *colonization. Forty-five patients were males. Their age ranged from 1 to 16 years. A child was considered as colonized if *Candida *sp. was isolated from one or more anatomic sites. A patient yielding *Candida *sp. on repeat cultures at least from one site was considered as persistently colonized [15]. The study was approved by the Ethics Committees of the Faculty of Medicine, Kuwait University and Ministry of Health, Kuwait. Informed consent of the patients was obtained before collecting the clinical samples.

### Isolation and identification

A total of 1075 swabs originating from oropharynx (n = 294), nasal (n = 600), rectum (n = 28), groin (n = 50), ear (n = 54), and axilla (n = 49) of 63 pediatric cancer patients were cultured on Sabouraud dextrose agar supplemented with chloramphenicol (Table 1). The germ tube test was performed on all the *Candida *spp. isolates for the presumptive identification of *C. albicans *or *C. dubliniensis*. Subsequently, *Candida *isolates were also identified by Vitek2 yeast identification system (BioMerieux, France). The identification of *Candida *spp. isolates was also confirmed by species-specific amplification and/or sequencing of internally transcribed spacer (ITS) region of rDNA.

**Table 1:** Surveillance cultures for yeast species in pediatric cancer patients

Sample site	No. positive/No. samples (%)
Oropharyngeal	53/294 (18)
Nasal	6/600 (1)
Rectal	10/28 (35.7)
Groin	5/50 (10)
Ear	1/54 (1.9)
Axilla	0/49 (0)
Total	75/1075 (7)

### Collection of serum samples

Five ml of blood was collected in sterile BDG-free clotting tubes and serum was separated for the detection of (1-3)-β-D-glucan (BDG), *Candida *mannan and species-specific *Candida *DNA at the time of surveillance culture. The serum was kept frozen at -20°C until used. Thirty-six serum samples from 20 colonized patients and 11 serum samples from nine non-colonized patients were tested.

### (1-3)-β-D-glucan detection in serum

The BDG levels in serum samples were determined using a Fungitell kit (Associates of Cape Cod Inc., East Falmouth, MA, USA) according to the procedure described by the manufacturer. BDG levels were assayed against a purified Pachyman standard, which included a five-point two-fold curve ranging from 31 pg/ml to 500 pg/ml. In brief, 5 μl of serum was dispensed per well in duplicate and pretreated with 20 μl of 0.25 M KOH and 1.2 M KCl for 10 min at 37°C. This step inactivated protease and other inhibitors present in human serum. The Fungitell BG reagent was then reconstituted and dispensed according to the instructions supplied by the manufacturer of the Fungitell kit. A Microplate Spectrphotometer (Bio-Tek Instruments, Inc., Winooski, VT, USA) with Gen5™ software onboard was used to accomplish kinetic analysis of the microtiter plate. The BDG value of ≥80 pg/ml was considered as positive and a value between 60-79 pg/ml as intermediate.

### *Candida *mannan detection in serum

Mannan antigen was measured by Platelia *Candida *Ag (BioRad, Marnes La Coquette, France). The test was performed according to the instructions of the manufacturer. Briefly, each test serum (300 μl) was mixed with 100 μl of treatment solution and placed in a boiling water bath for 3 minutes. After centrifugation, the supernatant was used for further testing. Fifty-μl of the conjugate and an equal amount of the treated serum supernatant was introduced into micro-titer plate wells pre-coated with anti-mannan monoclonal antibody. After incubation at 37°C for 90 min and 5 washing steps, 200 μl of the substrate buffer was added to each well, and the plates were incubated for 30 min at room temperature. The enzymatic reaction was terminated by adding stopping solution and the optical density was read at 450 nm using a Microplate Spectrphotometer (Bio-Tek Instruments, Inc.). The reactions were performed in duplicates and each experiment included positive and negative controls as well as a calibration curve. The calibration curve was made with a pool of normal human serum supplemented with known concentrations of mannan ranging from 0.1 to 2 ng/ml. A value of ≥0.5 ng/ml was taken as positive and ≥0.25 ng/ml but ≤0.5 ng/ml as doubtful.

### *Candida *DNA detection by PCR

DNA from cultured *Candida *spp. was isolated as described in detail previously [16,17]. DNA from serum was extracted using the QIAamp DNA kit (QIAGEN, Hilden, Germany) by following the instructions supplied by the manufacturer. DNA sequences of pan-fungal and species-specific forward and reverse primers and DNA amplification protocol were same as described previously [16,18]. Cultures of *C. albicans *ATCC 90029, *C. parapsilosis *ATCC 10233, *C. tropicalis *ATCC 750, *C. glabrata *ATCC 90030 and *C. dubliniensis *CBS 7987/CD36 were used as reference for amplification of specific products. The amplified DNA fragments were detected by agarose gel electrophoresis using 2% agarose gels as described previously [19].

The DNA isolated from selected isolates was also subjected to direct DNA sequencing of ITS region of rDNA (containing the ITS-1, 5.8S rRNA and ITS-2) to confirm species-specific identification by PCR. The ITS region was amplified by using ITS1 and ITS4 primers and both strands of amplified DNA were sequenced as described previously [20,21]. The sequencing primers, in addition to the amplification primers, included ITS1FS, ITS2, ITS3 and ITS4RS and the sequences were assembled as described previously [22]. GenBank basic local alignment search tool (BLAST) searches (http://www.ncbi.nlm.nih.gov/BLAST/Blast.cgi?) were performed for species identification

### Statistical analysis

Mann-Whitney 2-tailed test was applied to determine the significance of differences that existed between BDG or *Candida *mannan levels between different patient groups who were colonized with *Candida *spp. on single occasion or persistently and non-colonized subjects. The Spearman correlation test was performed to determine the correlation between BDG and *Candida *mannan. A *P *value of <0.05 was considered as significant.

## Results

### Surveillance cultures

The results of the surveillance cultures for *Candida *spp. are presented in Tables 1-2. Of 1075 swabs cultured from different anatomic sites of 63 pediatric cancer patients, 75 (7.8%) were positive for *Candida *spp. These isolates included *C. albicans *(n = 62), *C. dubliniensis *(n = 8), *C. glabrata *and *C. tropicalis *(n = 2 each) and *C. krusei *(n = 1) (Tables 1-2). Apart from phenotypic identification, all *Candida *spp. isolates were also identified by species-specific amplification of ITS region of rDNA (data not shown). The identity of six selected isolates was further confirmed by direct DNA sequencing of ITS region of rDNA (EMBL Accession Nos. FN652297, FN652298, FN652301 to FN652304). The distribution of anatomic sites yielding *Candida *spp. in culture was as follows: rectum, 36%; oropharynx, 18%; groin, 10%; ear, 2% and nasal, 1% (Table 1). Seventeen patients were colonized at one site and 18 at two or more sites. None of the swabs taken from axilla were positive for *Candida *spp. Of the 8 *C. dubliniensis *isolates, 5 came from oropharynx, and one each from nose, groin and rectum of six patients. Two patients yielded *C. glabrata *(nose and rectum) and two others yielded *C. tropicalis *(oropharynx and rectal). A single isolate of *C. krusei *was recovered from the oropharynx (Table 2).

**Table 2:** Species spectrum of *Candida *species isolated from different anatomic sites of pediatric cancer patients

Site	*C. albicans*	*C. dubliniensis*	*C. glabrata*	*C. tropicalis*	*C. krusei*
Oropharyngeal	46	5	0	1	1
Nasal	3	1	1	0	0
Rectal	8	1	1	1	0
Groin	4	1	0	0	0
Ear	1	0	0	0	0
Total	62	8	2	2	1

In colonized patients, the mean *Candida *mannan value was 0.16 ± 0.044 ng/ml, which was not significantly different from those that were not colonized (*p *= 0.660) (Table 3). There was also no significant difference in the mean *Candida *mannan values between patients colonized at one anatomic site or two or more sites (p = 0.665) or between those that were colonized once or those yielding *Candida *spp. repeatedly (*p *= 0.474) (Table 3). Although mean BDG values in colonized patients were nearly same as non-colonized patients (46.98 pg/ml vs. 36.77 pg/ml), 8 serum samples from 6 colonized patients were positive for BDG (range 82 pg/ml to 141 pg/ml, mean = 98.3 pg/ml). Three of these serum samples were obtained from the same patient within a span of 40 days and the BDG levels varied between 85 pg/ml to 115 pg/ml. However, none of these 8 serum samples yielded mannan levels >0.25 ng/ml. Additionally, 5 serum samples from two colonized patients yielded BDG values in the intermediate range (Table 3). There was no significant difference in the mean BDG values between patients that were colonized once or those that yielded *Candida *spp. persistently (*p *= 1.0) (Table 3). The mean and standard deviation of BDG and *Candida *mannan levels in serum samples collected from the patients at the time of surveillance samples were 42.74 ± 30.18 pg/ml and 0.173 ± 0.03 ng/ml, respectively. No correlation was observed between BDG and *Candida *mannan values among patients that were colonized with *Candida *species by Spearman correlation test (*p *= 0.531, *R *= 0.108). None of the serum sample from the colonized patients was positive for the detection of *Candida *DNA by PCR.

**Table 3:** (1-3)-β-D-glucan and *Candida *mannan levels in serum samples of different groups of patients

Colonization Status	No. of patients	No. of samples	BDG (pg/μl)			GM ± SD	Mannan (ng/ml)			GM ± SD
			**≥80**	**<80 - ≥60**	**<60**		**≥0.5**	**<0.5 - ≥0.25**	**<0.25**	
**Colonized**	20	36	8	5	23	46.98 ± 30.22	-	2	34	0.166 ± 0.044
**Persistently colonized**	10	20	3	5	12	46.60 ± 29.30	-	1	19	0.169 ± 0.046
**One-time colonized**	10	16	5	-	11	47.45 ± 32.30	-	1	15	0.164 ± 0.044
**Non-colonized**	9	11	-	-	11	36.77 ± 9.96	-	1	10	0.161 ± 0.06

GM, Geometric mean; SD, Standard deviation

**Figure 1:**
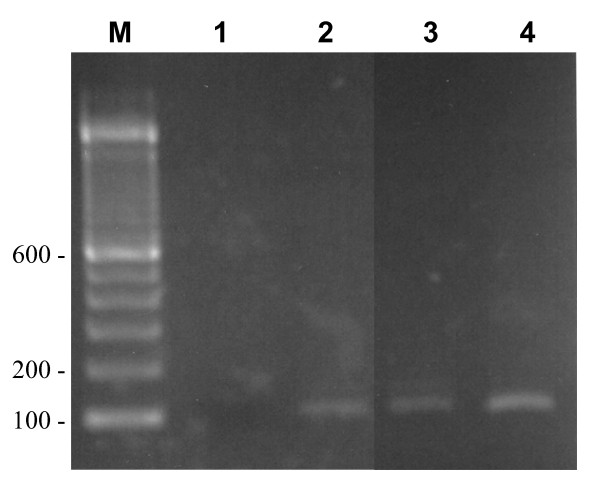
**Agarose gel showing amplification of a DNA fragment of ~106 bp by seminested PCR with DNA isolated from serum from patient 1 (lane 2) and patient 2 (lane 3)**. An amplicon of the same size was also obtained with genomic DNA isolated from reference strain of *C. tropicalis *(lane 4) while no amplicon was obtained in the reagent control tube in which water instead of DNA was added (lane 1). Lane M is 100 bp DNA marker and the positions of migration of 100 bp, 200 bp and 600 bp fragments are marked.

Two patients whose oropharyngeal or rectal samples yielded *C. tropicalis *(in addition to *C. dubliniensis *in one and *C. albicans *in the other) developed candidemia due to *C. tropicalis. *Besides blood culture positivity, both these patients showed presence of *C. tropicalis *DNA in serum by species-specific PCR amplification (Fig. 1) and also showed elevated levels of BDG (238.6 and 406 pg/ml), whereas *Candida *mannan was positive in one (0.77 ng/ml) and border line (0.28 ng/ml) in the other.

## Discussion

Infections caused by *Candida *spp. are the major cause of morbidity and mortality among seriously ill patients. Prior colonization with *Candida *spp. has been regarded as an essential step for the development of invasive disease [10,23]. The colonization index could be helpful in predicting risk of developing systemic infection in critically ill patients, and thus, may offer opportunities for early therapeutic or prophylactic interventions [6,10]. In the present study, although 11 of 35 (31%) patients were colonized with *Candida *spp. At ≥3 sites, none of them, despite being leukemic, developed candidemia or invasive candidiasis. Two patients with ALL who subsequently developed candidemia due to *C. tropicalis *were colonized at two sites with two different *Candida *spp. (*C. albicans *with *C. tropicalis *or *C. dubliniensis *with *C. tropicalis*) and their serum samples also yielded positive results for *C. tropicalis *DNA, mannan and BDG. Recently, Leon *et al. *[9] conducted a prospective observational study in a cohort of non-neutropenic patients to assess the value of "*Candida *score" for the probability of developing invasive candidiasis. Since invasive candidiasis occurred only in <5% of patients who had *Candida *colonization score of <3, the likelihood of developing invasive candidiasis in such patients was considered very low.

Since early diagnosis of invasive candidiasis is challenging, the role of surrogate markers, such as *Candida *species-specific DNA, mannan, and BDG in predicting the onset of invasive candidiasis has attracted considerable attention [13,24]. None of the colonized patients in the present study were found positive for *Candida *mannan or *Candida *DNA. However, BDG levels were positive in eight serum samples from six patients with values ranging from 82 pg/ml to 141 pg/ml. These observations are generally in agreement with previous studies showing that patients colonized at single or multiple sites yield BDG levels below the cut-off value recommended by the manufacturer [12,25-27]. The BDG levels above the cut-off values (80 pg/ml) in 6 of 20 (30%) colonized pediatric cancer patients in our study may either result from absorption of BDG through the gut due to mucositis [24] or due to contamination with cellulose [28], gauze [29], bacterial sepsis [30,31] or intravenous therapy with amoxicillin-calvulanic acid in these subjects [32]. Despite the above limitations of the test, several studies have used BDG monitoring to identify patients at risk of developing invasive candidiasis to improve therapeutic outcome [25,33,34]. A wide range of sensitivities and specificities have been obtained in different study populations [25,34,35], probably due to use of different cut-off values for a positive BDG test or due to use of different brand of kits that may react differently to BDG present in the clinical samples [36,37]. Unlike BDG, *Candida *mannan levels in serum seem to be less susceptible to the extent of *Candida *colonization. As stated above, none of our colonized patient was found to have positive serum levels (>0.5 ng/ml) for *Candida *mannan (mean 0.16 ± 0.04 ng/ml). In two patients who had *Candida *mannan in the intermediate range (0.308 and 0.287 ng/ml), sera were negative for BDG as well as *Candida *DNA. These findings are in conformity with the results of previous studies [12,14].

The normal BDG values in healthy pediatric population are not yet established. In a preliminary study, Brian Smith et al. [38] estimated BDG levels in serum samples from 120 non-immunocompromised children (0.5 to 18 year-old) and found higher levels (mean 68 ± 128 pg/ml) than those reported earlier in adult population [25,35]. A noteworthy observation of this study was that 18 of 120 (15%) children had BDG levels >80 pg/ml and 8 of 120 (7%) had BDG levels between 60-79 pg/ml [38]. Thus, higher BDG levels in a minority (6 of 20, 30%) of pediatric cancer patients in our study is consistent with the BDG data reported by Brian Smith et al. [38]. These observations warrant further studies in pediatric population for establishing BDG cut-off values for a positive test to validate the diagnostic utility of this marker for invasive fungal infections.

## Conclusions

This prospective surveillance study revealed that while nearly half (55%) of the pediatric cancer patients were colonized with *Candida *spp. at one or more anatomic sites, the serum levels for *Candida *mannan remained significantly less than the cut-off value recommended by the manufacturer for a positive test. Also, *Candida *DNA was not detected in the serum samples of the colonized patients by the semi-nested PCR used. However, BDG levels were elevated in a minority of colonized pediatric patients suggesting that additional evidence of infection may be needed for the diagnosis of invasive candidiasis in this patient population.

## Abbreviations

BDG: (1-3)-β-D-glucan; PCR: Polymerase Chain Reaction; DNA: Deoxyribonucleic Acid; ALL: Acute Lymphoblastic Leukemia; AML: Acute Myeloblastic leukemia; KOH: Potassium Hydroxide; KCl: Potassium Chloride; ITS: Internally Transcribed Spacer; CBS: Centraalbureau voor Schimmeculture

## Competing interests

The authors declare that they have no competing interests.

## Authors' contributions

EM, MHB, and ZUK: Conceived and supervised the study. SA supervised the molecular part of the study. All the authors contributed to the writing and finalizing the manuscript.

## Pre-publication history

The pre-publication history for this paper can be accessed here:

http://www.biomedcentral.com/1471-2334/10/292/prepub
